# Optical coherence tomography findings after drug-coated balloon treatment for *de novo* coronary artery lesions with eruptive calcified nodule

**DOI:** 10.3389/fcvm.2025.1666458

**Published:** 2025-11-13

**Authors:** Kazuki Matsuda, Tetsumin Lee, Takashi Ashikaga, Toshihiro Nozato, Yasutoshi Nagata, Masakazu Kaneko, Ryoichi Miyazaki, Toru Misawa, Masashi Nagase, Tomoki Horie, Mao Terui, Daigo Kachi, Yuki Odanaka, Maki Ohira, Naoya Kikkoji, Ayaka Koido, Megumi Kou, Risako Baba, Akira Takakuma, Taishi Yonetsu, Tetsuo Sasano

**Affiliations:** 1 Department of Cardiology, Japanese Red Cross Musashino Hospital, Tokyo, Japan; 2 Department of Cardiovascular Medicine, Institute of Science Tokyo, Tokyo, Japan

**Keywords:** drug-coated balloon, calcified nodule, optical coherence tomography, target-lesion failure, target-lesion revascularization, coronary artery disease

## Abstract

The purpose of the present study was to investigate the factors associated with worse clinical outcomes in patients with *de novo* eruptive calcified nodule (CN) lesions after stent-less percutaneous coronary intervention (PCI) with a drug-coated balloon (DCB), as assessed by optical coherence tomography (OCT) and morphological findings at target lesion revascularization (TLR) or during the follow-up period. We retrospectively enrolled 68 *de novo* eruptive coronary artery eruptive CN lesions in 58 patients who underwent PCI with DCB. All lesions were treated without stents and underwent both pre- and post-PCI OCT. The patients were divided into two groups: those with or without target lesion failure (TLF), defined as a composite of culprit lesion-related cardiac death, myocardial infarction, and TLR. At a median follow-up period of 650 days, TLF events occurred in 14 lesions (20.6%) and were associated with the absence of medial involvement of coronary artery dissection on post-PCI OCT (28.6% vs. 70.4%, *P* = 0.006). In the subgroup analysis of 16 lesions with serial OCT imaging (pre-and post-PCI at the index PCI and at TLR or follow-up), TLR occurred in nine lesions. We found CN protrusion at TLR in seven lesions, layered plaque at TLR in one lesion, and suboptimal lumen expansion at the index PCI in another lesion as restenosis patterns. Moreover, CN protrusion was significantly more frequent in TLR lesions than in the seven non-TLR lesions (77.8% vs. 14.3%, *P* = 0.041). In conclusion, we observed a high incidence of TLF after DCB treatment for *de novo* eruptive CN coronary artery lesions, which correlated with the absence of medial involvement in dissection. CN protrusion is frequently observed in TLR lesions, whereas late lumen enlargement is predominantly observed in non-TLR lesions.

## 1 Introduction

Severely calcified coronary lesions are challenging to treat with percutaneous coronary intervention (PCI) using drug-eluting stents (DES), and they cause stent under-expansion even if scoring/cutting balloons or atherectomy devices are used (1). Among several calcified plaque characteristics, calcified nodules (CN) have been reported to be associated with poor prognosis (2–5) after DES implantation. Recently, PCI using a drug-coated balloon (DCB) without a stent has been reported to be non-inferior to DES; it is considered an alternative option (6, 7) and is currently used in regular practice (8). Mitsui et al. reported that DCB was comparable to DES in patients with severely calcified plaques requiring orbital atherectomy (9). However, there are limited data regarding the clinical outcomes of *de novo* CN lesions after stent-less PCI with DCB.

Recently, optical coherence tomography (OCT) revealed predictors of target lesion revascularization (TLR) after DES implantation for CN (10). OCT can identify eruptive CNs, which differ from non-eruptive CNs in terms of mechanism and prognostic significance (4). Additionally, unique underlying morphological findings have been reported in TLR lesions after DCB treatment (11). However, the predictors and morphological changes associated with restenosis following DCB treatment for eruptive CN lesions have not been fully clarified.

Thus, the purpose of the present study was to investigate factors associated with worse clinical outcomes in patients with *de novo* eruptive CN lesions after stent-less PCI with DCB as assessed by OCT and morphological findings at TLR or during the follow-up period.

## 2 Materials and methods

### 2.1 Study design, patient population, and endpoint

**Figure 1:**
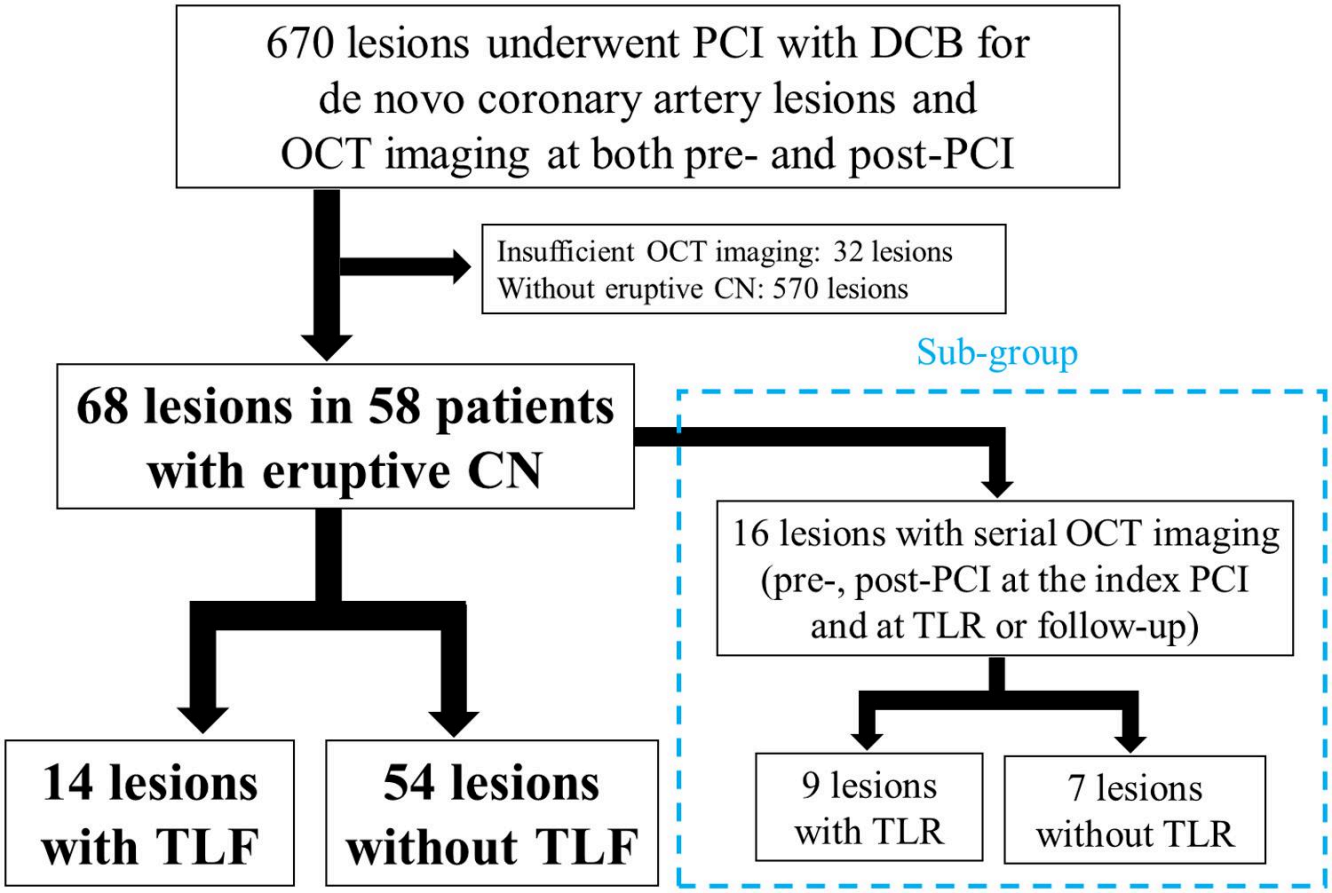
Study flow chart. Of the 670 *de novo* coronary artery lesions that were treated with OCT-guided PCI with DCB, 68 lesions were enrolled from 58 patients with eruptive CN, and TLF events occurred in 14 lesions. In the subgroup of 16 lesions with serial OCT imaging (pre-and post-PCI at the index PCI and at TLR or follow-up), TLR occurred in nine lesions. PCI, percutaneous coronary intervention; DCB, drug-coated balloon; OCT, optical coherence tomography; CN, calcified nodule; TLF, target lesion failure; TLR, target lesion revascularization.

This retrospective observational study was conducted at the Japanese Red Cross Musashino Hospital (Tokyo, Japan). Between May 2018 and February 2024, 670 *de novo* coronary artery lesions were treated with stent-less PCI with DCB and both pre- and post-PCI OCT imaging; 68 eruptive CN lesions in 58 patients were enrolled in the present study (Figure 1). The exclusion criteria were as follows: (1) DES implantation at the index PCI, (2) in-stent restenosis, (3) coronary artery bypass grafted lesions, and (4) insufficient OCT imaging. The patients were divided into two groups: those with or without target lesion failure (TLF), defined as a composite of culprit lesion-related cardiac death, nonfatal myocardial infarction (MI), and clinically driven TLR. Clinical follow-up data were obtained during outpatient clinical visits and telephone interviews. Diagnosis of MI was based on the current universal definition of MI (12). TLR was defined as clinically driven repeat revascularization. Furthermore, a subgroup of 16 lesions with serial OCT imaging (pre-and post-PCI at the index PCI and at TLR or follow-up) was assessed to examine the morphological patterns of restenosis in eruptive CN lesions. The study was conducted in accordance with the Declaration of Helsinki and approved by the Institutional Ethics Committee (June 30, 2025, No. 7014). All patients provided written informed consent for future enrolment in institutional clinical studies. Prompt optimal medical therapy was initiated in all patients before PCI, and guideline-directed medical therapy was continued thereafter (13).

### 2.2 Coronary angiography and index PCI

During index PCI, the intervention was performed according to a recent expert consensus document (14). For optimal lesion preparation, rotational atherectomy, orbital atherectomy, intravascular lithotripsy, and excimer laser coronary atherectomy have been recommended (15, 16) and were performed at the operator's discretion. Additionally, balloon angioplasty, including semi-compliant balloon, scoring balloon, or cutting balloon before DCB procedure, was performed (recommended balloon to vessel ratio, 0.8–1.0). DES implantation was recommended in cases of flow-limiting dissection or classification of National Heart, Lung, and Blood Institute (NHBLI) type D-F dissection (17) after lesion preparation, and these patients were excluded. Paclitaxel-coated balloons (SeQuent Please; B. Braun Melsungen AG or AGENT; Boston Scientific, USA) were used as DCB devices.

Quantitative coronary angiography was performed using QCA-CMS (Medis Medical Imaging Systems, Leiden, Netherlands). The minimum lumen diameter, reference diameter, % diameter stenosis, and lesion length were measured in diastolic frames from orthogonal projections. Angiographic calcification at the target lesion site was classified as none, mild, moderate, or severe at the target lesion site (18). Moderate calcification was defined as a radiopacity noted only during the cardiac cycle before contrast injection, whereas severe calcification was defined as a radiopacity observed without cardiac motion, usually affecting both sides of the arterial lumen. Coronary artery dissection was assessed according to the NHBLI classification (17).

All patients were routinely treated with oral antiplatelet therapy, including aspirin, clopidogrel, or prasugrel, according to the Japanese Circulation Society guidelines (19).

### 2.3 OCT image acquisition and analysis

We used frequency-domain OCT (Dragonfly OPTIS or Opstar OCT imaging catheter; Abbott Vascular, Santa Clara, CA, USA) or a high-frequency OCT system (Gentuity Vis-Rx Micro-Imaging Catheter; Nipro), and all OCT images were analyzed using proprietary software based on previously validated criteria for OCT plaque characterization (20, 21).

We quantitatively and qualitatively evaluated the pre- and post-PCI OCT findings at the index PCI for all lesions and serial OCT findings at TLR or follow-up for subgroups. The minimum lumen area (MLA) and area stenosis were evaluated for all lesions. On pre-PCI OCT, the lipid arc was defined as the circumferential extent of lipid material imaged as a signal-poor region diffusely bordered by overlying signal-rich bands corresponding to a fibrous cap (22). Calcium level was defined as a signal-poor or heterogeneous region with a sharply delineated border. The maximum arc of the target lesion calcium was measured in degrees by using a protractor centered on the lumen. The maximum calcium thickness was also measured (23). The maximum eruptive CN arc angle was assessed in the frame displaying the greatest eruptive CN arc angle, not including calcified protrusion or calcific sheet, in the lesion. Eruptive CN length was measured from the proximal to distal ends within the lesion as reported previously (24). In this study, CN implied only eruptive CN, defined as a lesion with fibrous cap disruption and a luminal thrombus associated with eruptive dense calcific nodules (3, 25). Post-PCI OCT was used to assess the presence of coronary artery dissections (26). Axial injury of the dissection was described as intimal involvement of the coronary dissection, where only the intima was affected and the media was still intact, as medial involvement if it extended into the media without disruption of the entire medial layer, and as adventitial involvement when the media was dissected throughout its thickness. Additionally, the maximum and longitudinal dissection angles were assessed. Suboptimal lumen expansion was defined as lesions with both post-PCI MLA <3.00 mm^2^ and expansion ratio (post-PCI MLA/mean reference lumen area) <0.50 at the index PCI (11). In the subgroup analysis, we investigated the presence or absence of underlying plaque morphology, as reported previously (11): CN protrusion, layered plaque, thrombus, and residual coronary artery dissection at TLR or follow-up. A CN protrusion was defined as a calcified nodule protruding into the lumen (27) and a layered plaque was defined as a different optical intensity with a clear demarcation from the underlying plaque (28). Late lumen enlargement (LLE) was defined as the MLA being larger during the follow-up period than post-PCI at the index (29).

### 2.4 Statistical analysis

Statistical analyses were performed using R version 4.5.0 (The R Foundation for Statistical Computing, Vienna, Austria) and SPSS software (version 22.0; IBM, Armonk, New York, USA). Categorical data were expressed as numbers and frequencies and compared using *χ*^2^ or Fisher's exact test, as appropriate. As most values were not normally distributed, continuous variables were expressed as medians (interquartile range [IQR]) and compared using the Mann–Whitney *U* test. A two-sided *P* < 0.05 was considered statistically significant. Cox regression analyses were performed to identify predictors of TLF during the follow-up period. Hazard ratios with corresponding 95% confidence intervals are calculated. All variables associated with adverse events at *P* < 0.10 level in the univariate analysis were then tested in a multivariate Cox regression analysis. Intraobserver and interobserver differences were quantified using the kappa coefficient of agreement for the plaque classification. Statistical significance was set at *P* < 0.05.

## 3 Results

In the present study, 68 eruptive CN lesions were identified in 58 patients. TLF events occurred in 14 lesions (20.6%), including two cardiac deaths (2.9%), and seven non-fatal MI (10.3%) and 11 TLR (16.2%) cases. The median follow-up periods were 650 days (IQR: 393–919 days), and TLF occurred 293 days (IQR: 238–854 days) after the index PCI. Additionally, nine patients died due to non-cardiac reasons.

### 3.1 Baseline patient characteristics

The baseline characteristics of the patients are summarized in Table 1. The median patient age was 77 years (IQR: 69–81 years), and 70.7% of patients were male. The overall prevalence of chronic kidney disease and hemodialysis was high, with a more pronounced trend in the TLF group, although not statistically significant (estimated glomerular filtration rate: 11.9 [IQR: 5.2–74.3] mL/min/1.73 m^2^ vs. 50.6 [IQR: 13.1–64.4] mL/min/1.73 m^2^, *P* = 0.352; hemodialysis: 50.0% vs. 30.4%, *P* = 0.307). The most common clinical presentation at the index PCI was stable angina (65.5%). Patients who had previously undergone MI or PCI and those with a low ejection fraction had TLF (prior MI: 58.3% vs. 23.9%, *P* = 0.035; prior PCI: 83.3% vs. 43.5%, *P* = 0.022; ejection fraction: 44 [IQR: 37–61] % vs. 64 [IQR: 54–73] %, *P* = 0.015).

**Table 1:** Patient characteristics at the index PCI.

Variable	Total (*n* = 58)	Patients with TLF (*n* = 12)	Patients without TLF (*n* = 46)	*P* value
Age, (years)	77 (69–81)	72 (69–74)	79 (71–82)	0.080
Male, *n* (%)	41 (70.7%)	10 (83.3%)	31 (67.4%)	0.478
Hypertension, *n* (%)	49 (84.5%)	12 (100.0%)	37 (80.4%)	0.181
Diabetes mellitus, *n* (%)	30 (51.7%)	7 (58.3%)	23 (50.0%)	0.749
Dyslipidemia, *n* (%)	38 (65.5%)	8 (66.7%)	30 (65.2%)	1.000
Current smoker, *n* (%)	18 (31.0%)	3 (25.0%)	15 (32.6%)	0.736
eGFR (ml/min/1.73 m^2^)	48.6 (8.2–65.0)	11.9 (5.2–74.3)	50.6 (13.1–64.4)	0.352
Hemodialysis, *n* (%)	20 (34.5%)	6 (50.0%)	14 (30.4%)	0.307
Prior MI, *n* (%)	18 (31.0%)	7 (58.3%)	11 (23.9%)	0.035
Prior PCI, *n* (%)	30 (51.7%)	10 (83.3%)	20 (43.5%)	0.022
Prior CABG, *n* (%)	4 (6.9%)	0 (0.0%)	4 (8.7%)	0.571
Stable CAD, *n* (%)	38 (65.5%)	7 (58.3%)	31 (67.4%)	0.734
ACS, *n* (%)	20 (34.5%)	5 (41.7%)	15 (32.6%)	0.734
EF (%)	61 (44–72)	44 (37–61)	64 (54–73)	0.015
Duration of DAPT	144 (79–269)	468 (127–788)	135 (78–233)	0.036

PCI, percutaneous coronary intervention; TLF, target lesion failure; eGFR, estimated glomerular filtration rate; MI, myocardial infarction; CABG, coronary artery bypass graft; CAD, coronary artery disease; ACS, acute coronary syndrome; EF, ejection fraction; DAPT, dual antiplatelet therapy.

### 3.2 Angiographic and procedural findings at the index PCI

Table 2 shows angiographic and procedural findings at the index PCI in lesions with and without TLF. Calcific plaque modification devices before DCB treatment were required for 66 lesions (97.1%) (rotational atherectomy, 10 lesions [14.7%]; orbital atherectomy, 49 lesions [72.1%]; excimer laser, eight lesions [11.8%]; and intravascular lithotripsy, nine lesions [13.2%]). There were more TLF events in lesions with cutting balloons than in those with scoring balloons, but the difference was not significant. There were no significant differences in pre- and post-PCI angiographic findings and other PCI procedures between lesions with or without TLF.

**Table 2:** Angiographic and procedure results at the index PCI.

Variable	Total (*n* = 68)	Lesions with TLF (*n* = 14)	Lesions without TLF (*n* = 54)	*P* value
Target Vessel, *n* (%)				
RCA	31 (45.6%)	8 (57.1%)	23 (42.6%)	0.378
LAD	32 (47.1%)	5 (35.7%)	27 (50.0%)	0.383
LCX	5 (7.4%)	1 (7.1%)	4 (7.4%)	1.000
Calcification, *n* (%)				
None or mild	1 (1.5%)	0 (0%)	1 (1.9%)	1.000
Moderate	7 (10.3%)	1 (7.1%)	6 (11.1%)	1.000
Severe	60 (88.2%)	12–3 (92.9%)	47 (87.0%)	1.000
Pre-PCI QCA				
Minimum lumen diameter, mm	0.92 (0.68–1.28)	0.92 (0.84–1.09)	0.90 (0.62–1.31)	0.921
Reference vessel diameter, mm	2.67 (2.37–3.13)	2.85 (2.57–3.15)	2.64 (2.31–3.13)	0.339
Diameter stenosis, %	63.5 (52.1–78.2)	67.4 (55.6–75.0)	61.0 (51.1–78.2)	0.549
Lesion length, mm	14.6 (9.8–24.8)	9.8 (9.2–18.2)	15.3 (10.9–24.9)	0.065
Post-PCI Angiography findings				
Minimum lumen diameter, mm	2.05 (1.73–2.35)	2.10 (1.93–2.33)	2.02 (1.72–2.34)	0.606
Diameter stenosis, %	25.1 (18.7–31.3)	30.2 (24.5–35.7)	24.6 (18.6–30.3)	0.143
Acute gain, mm	1.03 (0.65–1.42)	1.03 (0.75–1.56)	1.01 (0.65–1.34)	0.468
Post-PCI dissection classification (NHLBI classification), *n* (%)				
Type A	11 (16.2%)	1 (7.1%)	10 (18.5%)	0.437
Type B	14 (20.6%)	2 (14.3%)	12 (22.2%)	0.717
Type C	3 (4.4%)	0 (0.0%)	3 (5.6%)	1.000
PCI procedure results				
Cutting balloon, *n* (%)	28 (41.2%)	9 (64.3%)	19 (35.2%)	0.069
Scoring balloon, *n* (%)	40 (58.8%)	5 (35.7%)	35 (64.8%)	0.069
Maximum balloon size, mm	3.0 (2.5–3.3)	3.1 (2.8–3.5)	3.0 (2.5–3.3)	0.112
Maximum inflation pressure, mmHg	16 (12–24)	14 (11–20)	16 (12–24)	0.412
Rotational atherectomy, *n* (%)	10 (14.7%)	2 (14.3%)	8 (14.8%)	1.000
Orbital atherectomy, *n* (%)	49 (72.1%)	9 (64.3%)	40 (74.1%)	0.512
Excimer laser, *n* (%)	8 (11.8%)	3 (21.4%)	5 (9.3%)	0.347
Intravascular lithotripsy, *n* (%)	9 (13.2%)	1 (7.1%)	8 (15.1%)	0.672
Total DCB length, mm	30 (20–47)	26 (20–30)	33 (20–50)	0.063
Maximum DCB diameter, mm	3.0 (3.0–3.5)	3.5 (3.0–3.9)	3.0 (3.0–3.5)	0.131
Maximum inflation pressure of DCB, mmHg	6 (6–7)	7 (6–7)	6 (6–7)	0.355

PCI, percutaneous coronary intervention; TLF, target lesion failure; RCA, right coronary artery; LAD, left anterior descending artery; LCX, left circumflex artery; QCA, quantitative coronary angiography; NHLBI, National Heart, Lung, and Blood Institute; DCB, drug-coated balloon.

### 3.3 OCT findings at index PCI

Table 3 shows OCT findings at the index PCI in lesions with or without TLF. There were no significant differences in lipid arc and calcium severity at pre-PCI. A larger eruptive CN angle correlated with TLF (157° [IQR: 122–225] vs. 115° [80–155], *P* = 0.016). In post-PCI OCT findings, medial involvement of coronary artery dissection was less frequent (28.6% vs. 70.4%, *P* = 0.006), and dissection length was shorter in patients with TLF than in those without (6.0 mm [IQR: 5.4–13.0] vs. 12.4 mm [8.4–23.8], *P* = 0.017), whereas the angle of non-calcified area at MLA was similar between lesions with or without TLF (74° [IQR: 0–142] vs. 91° [28–250], *P* = 0.422). Post-PCI MLA was similar between the two groups. Favorable intraobserver and interobserver agreement was observed in the diagnosis of each subtype of calcified culprit plaque (kappa = 0.897 and 0.941, respectively).

**Table 3:** OCT findings.

Variable	Total (*n* = 68)	Lesions with TLF (*n* = 14)	Lesions without TLF (*n* = 54)	*P* value
Pre-PCI OCT findings				
Pre-PCI minimum lumen area, mm^2^	1.68 (1.15–2.30)	1.64 (1.03–2.44)	1.68 (1.20–2.25)	0.649
Maximum calcium angle,°	360 (256–360)	360 (239–360)	360 (257–360)	0.708
Maximum calcium thickness, µm	1,160 (1,050–1,263)	1,205 (1,143–1,300)	1,150 (1,050–1,250)	0.264
Calcium length, mm	25 (20–34)	26 (19–30)	25 (21–34)	0.302
Maximum eruptive CN angle,°	123 (85–161)	157 (122–225)	115 (80–155)	0.016
Eruptive CN length, mm	4.2 (2.7–7.0)	5.3 (3.2–7.5)	4.0 (2.6–6.9)	0.419
Post-PCI OCT findings				
Post-PCI minimum lumen area, mm^2^	4.11 (3.33–4.74)	4.43 (3.32–4.73)	4.07 (3.38–4.72)	0.622
Post-PCI area stenosis, %	39.2 (26.4–48.7)	37.0 (31.7–52.6)	39.9 (25.3–48.5)	0.529
Post-PCI suboptimal lumen expansion, *n* (%)	6 (8.8%)	1 (7.1%)	5 (9.3%)	1.000
Post-PCI dissection				
None or Intimal involvement, *n* (%)	12 (17.6%)	8 (57.1%)	4 (7.4%)	<0.001
Medial involvement, *n* (%)	42 (61.8%)	4 (28.6%)	38 (70.4%)	0.006
Adventitial involvement, *n* (%)	14 (21.7%)	2 (14.3%)	12 (22.2%)	0.717
Maximum dissection angle,°	102 (67–153)	92 (66–132)	110 (71–158)	0.260
Longitudinal dissection length, mm	11.8 (6.6–21.7)	6.0 (5.4–13.0)	12.4 (8.4–23.8)	0.017

OCT, optical coherence tomography; TLF, target lesion failure; PCI, percutaneous coronary intervention; CN, calcified nodule.

### 3.4 Predictors of target lesion-related clinical event

Table 4 shows univariable and multivariable Cox regression analysis to predict TLF. In multivariable Cox regression analysis, history of prior PCI (hazard ratio: 5.70, 95% confidence interval: 1.20–27.1, *P* = 0.028) and absence of medial involvement of coronary artery dissection at the index PCI (hazard ratio: 0.21, 95% confidence interval: 0.06–0.72, *P* = 0.013) remained independent predictors of TLF.

**Table 4:** Univariable and multivariable Cox regression analysis of predictive factors of TLF.

Variable	Univariable Cox regression			Multivariable Cox regression		
	HR	95% CI	*P* value	HR	95% CI	*P* value
Age, (years)	0.98	0.93–1.03	0.431			
Prior PCI, *n* (%)	5.68	1.26–25.62	0.024	5.70	1.20–27.1	0.028
EF (%)	0.96	0.93–1.00	0.056	0.98	0.94–1.02	0.289
Lesion length on QCA, mm	0.96	0.90–1.02	0.182			
Cutting balloon use, *n* (%)	1.64	0.54–5.01	0.386			
Medial involvement of coronary dissection, *n* (%)	0.20	0.06–0.63	0.006	0.21	0.06–0.72	0.013

TLF, target lesion failure; HR, hazard ratio; CI, confidence interval; PCI, percutaneous coronary intervention; EF, ejection fraction; QCA, quantitative coronary angiography.

### 3.5 Sub-group of serial OCT imaging at TLR or follow-up

**Figure 2:**
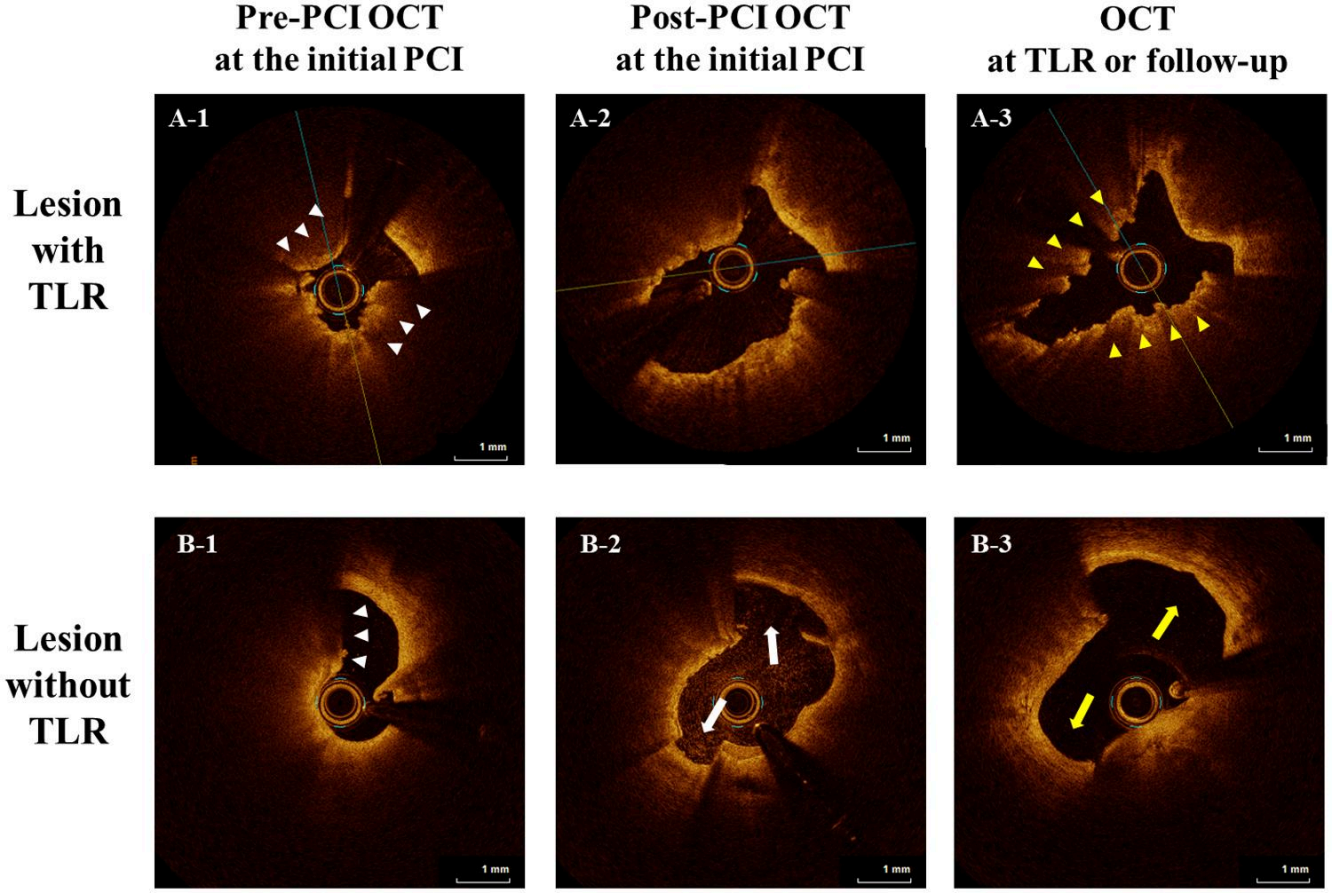
Representative serial images with and without TLR. Representative serial OCT images (pre-and post-PCI at the index PCI and TLR or the follow-up period) are shown. In both cases, calcified nodules (CN; white arrowheads) are observed in pre-PCI OCT images of the culprit lesion at the initial PCI **(A-1, B-1)**. In the upper case **(A-1,2,3)**, in the post-PCI image at the initial PCI, an adequate lumen area was obtained, but the dissection only extended to the intima **(A-2)**, and TLR with CN protrusion (yellow arrowheads) eventually occurred **(A-3)**. In contrast, in the lower case **(B-1,2,3)**, dissection of the medial involvement (white arrow) was observed in the post-PCI image at the initial PCI **(B-2)**, and they healed (yellow arrow) during the follow-up period **(B-3)**. OCT, optical coherence tomography; TLR, target lesion revascularization; PCI, percutaneous coronary intervention; CN, calcified nodule.

In a subgroup of 16 lesions with serial OCT imaging (pre- and post-PCI at the index PCI and at TLR or follow-up), TLR occurred in nine lesions and MLA at TLR or follow-up were 1.95 mm^2^ (IQR; 1.78–2.54) and 3.85 (IQR; 3.67–5.48), respectively (*P* = 0.004) (Table 5). Of the nine lesions treated with TLR, we identified seven CN protrusions, one layered plaque, and one suboptimal lumen expansion at the index PCI as restenosis patterns. Moreover, compared to the seven non-TLR lesions, CN protrusion was significantly more frequent in TLR lesions at TLR or during the follow-up period (77.8% vs. 14.3%, *P* = 0.041). LLE was found in six non-TLR lesions (85.7%) and absent in TLR lesions (*P* < 0.001). Representative serial images with and without TLR are shown in Figure 2 and images of the three main mechanisms of TLR are shown in Supplementary Figure.

**Table 5:** OCT findings at TLR or follow-up.

Variable	Total (*n* = 16)	Lesions with TLR (*n* = 9)	Lesions without TLR (*n* = 7)	*P* value
Minimum lumen area at TLR, mm^2^	3.19 (1.94–4.12)	1.95 (1.78–2.54)	3.85 (3.67–5.48)	0.004
Late lumen enlargement, *n* (%)	6 (37.5%)	0 (0.0%)	6 (85.7%)	<0.001
Residual dissection, *n* (%)	2 (12.5%)	1 (11.1%)	0 (0.0%)	0.475
Calcified nodule protrusion, *n* (%)	8 (50.0%)	7 (77.8%)	1 (14.3%)	0.041
Layered plaque, *n* (%)	1 (6.3%)	1 (11.1%)	0 (0.0%)	1.000

OCT, optical coherence tomography; TLR, target lesion revascularization.

## 4 Discussion

In this study, we investigated factors associated with worse clinical outcomes in patients with *de novo* eruptive CN lesions after stent-less PCI with DCB as assessed by OCT, as well as morphological findings at TLR or during the follow-up period. The main findings of this study are summarized as follows: (1) the prevalence of TLF after treatment with DCB for *de novo* eruptive CN lesions was 20.6%; (2) a history of prior PCI and the absence of medial involvement of coronary dissection at the time of the index PCI were associated with TLF; (3) regarding the morphological findings of TLR, CN protrusion was the most frequently found, followed by layered plaque and suboptimal lumen expansion; and (4) CN protrusion was significantly more frequent in TLR lesions than in non-TLR lesions. To the best of our knowledge, this is the first study to assess the eruptive CN at the *de novo* coronary artery lesions using serial OCT after PCI with DCB.

Various studies have assessed the morphology of calcified plaques. As reported in a pathological study (30), the progression of calcification typically starts with microcalcifications, which grow into larger fragments, forming spotty calcifications and eventually diffuse sheet-like deposits. Sheet calcifications may fracture, leading to the formation of nodular calcifications that protrude into the lumen or media (31). According to previous reports, calcified plaques identified using OCT can be classified into three types: eruptive CN, superficial calcific sheets, and calcified protrusions (32, 33).

Among the severely calcified plaques, intervention for eruptive CN lesions remains challenging (2). CN has a unique plaque morphology in which an area of nodular calcification causes disruption of the fibrous cap with an overlying luminal thrombus (3). In a previous report, compared to non-eruptive CN lesions, eruptive lesions had worse post-stent long-term clinical outcomes despite better acute stent expansion (4) and CN lesions requiring rotational atherectomy were associated with a higher TLR rate within 1 year (5). Hamana et al. reported that factors such as younger age, hemodialysis, eruptive CNs, dark CNs, disrupted fibrous tissue, and irregular protrusions were independently related to TLR in patients with CNs treated with DES (10). Moreover, one study reported that a large eruptive CN angle was one of the predictors of TLF, which is in line with our results (24).

However, there are few previous studies on the DCB treatment of eruptive CN lesions (34). We found an association between the absence of medial involvement on post-PCI OCT and the frequency of TLF after DCB treatment of eruptive CN lesions. In previous reports including non-CN lesions, post-PCI coronary artery dissection on OCT was positively associated with LLE and fewer TLF events after DCB treatment (29, 35, 36). These findings are consistent with those of the present study. Furthermore, consistent with a previous study, there was a trend toward better outcomes in lesions with scoring balloons than in those with cutting balloons (37); however, no significant difference was found regardless of the calcific plaque modification device type (38). Moreover, in the multivariate analysis, a history of PCI was also correlated with TLF. A previous pathological study showed that a relatively greater total plaque burden was found in patients with eruptive CN, not only at the culprit but also in the non-culprit segment (3). This suggests that plaque progression may occur more frequently or repeatedly in patients with a history of PCI. Although the precise mechanism is unclear, a previous OCT study reported that prior MI and prior stenting were more frequently observed in CN lesions with TLR than in those without (10), which is in line with the present study. In this study, TLF group patients were tend to have poorer renal function and a higher proportion of dialysis. Hemodialysis is a known predictor of accelerated atherosclerosis and poor prognosis with CN lesions (39), and this factor may have an impact on the result of this study.

Similar to previous reports on lesions with DES implantation, serial OCT assessment in the present study revealed that CN plaques in the *de novo* coronary artery lesions could protrude at the TLR, even after stent-less PCI with DCB treatment at the index PCI. One plausible hypothesis is that TLR eventually occurs when the disadvantages of DCB treatment, such as the progression of CN protrusion, exceed the therapeutic benefits of LLE. Although establishing a definitive mechanism for TLR after DCB treatment remains challenging, calcium deposits may serve as a barrier to optimal drug absorption as reported previously (40). Therefore, adequate preparation for non-flow-limiting coronary dissection during index PCI might be an important factor in mitigating the aforementioned limitations. Further studies are required to determine the optimal treatment for eruptive CN lesions with DCBs.

### 4.1 Clinical implications

Percutaneous coronary intervention using DES has been recognized as a significant challenge in managing eruptive CN lesions. Moreover, DCB is associated with an unfavorable prognosis. The present study suggests that adequate lesion preparation, such as sufficient lumen enlargement with the dissection of medial involvement, could potentially contribute to the prevention of TLF when performing stent-less PCI with DCB for eruptive CN lesions.

### 4.2 Limitations

The present study had some limitations. First, in this retrospective observational study with both small and non-small vessel disease, the PCI procedure, including the selection of the DCB size and length, was determined at the operator's discretion, making selection bias unavoidable. Second, the sample size, especially of sub-analysis group, was small and the predictor analysis risks may be misinterpretation and overfitting. Third, lesions with anticipated difficulties in advancing the OCT catheter, such as those with severe narrowing, tortuosity, or calcification, were excluded, potentially affecting the applicability to more complex lesions. Fourth, the cases with bailout stenting as flow-limiting dissection or classification of NHBLI type D-F dissection are excluded. Fifth, the classification of TLR morphology was based on a limited number of OCT imaging cases and lacked pathological assessment. Thus, the classification of TLR morphology, including CN protrusion, is ambiguous. Further large-scale clinical studies and pathological assessments are warranted to elucidate the mechanisms underlying TLR in patients undergoing stent-less PCI with DCB for eruptive CN lesions.

## 5 Conclusion

We observed a high incidence of TLF after DCB treatment for *de novo* eruptive CN coronary artery lesions, which was correlated with the absence of medial coronary artery dissection. CN protrusions were frequently identified in TLR lesions, whereas LLE were predominantly observed in non-TLR lesions.

## Data Availability

The raw data supporting the conclusions of this article will be made available by the authors, without undue reservation.
