# Supplementary figures and images for: Optical coherence tomography findings after drug-coated balloon treatment for de novo coronary artery lesions with eruptive calcified nodule
Source: Front Cardiovasc Med. 2025 Nov 13;12:1666458. doi: 10.3389/fcvm.2025.1666458 (PMC12659186; doi:10.3389/fcvm.2025.1666458)

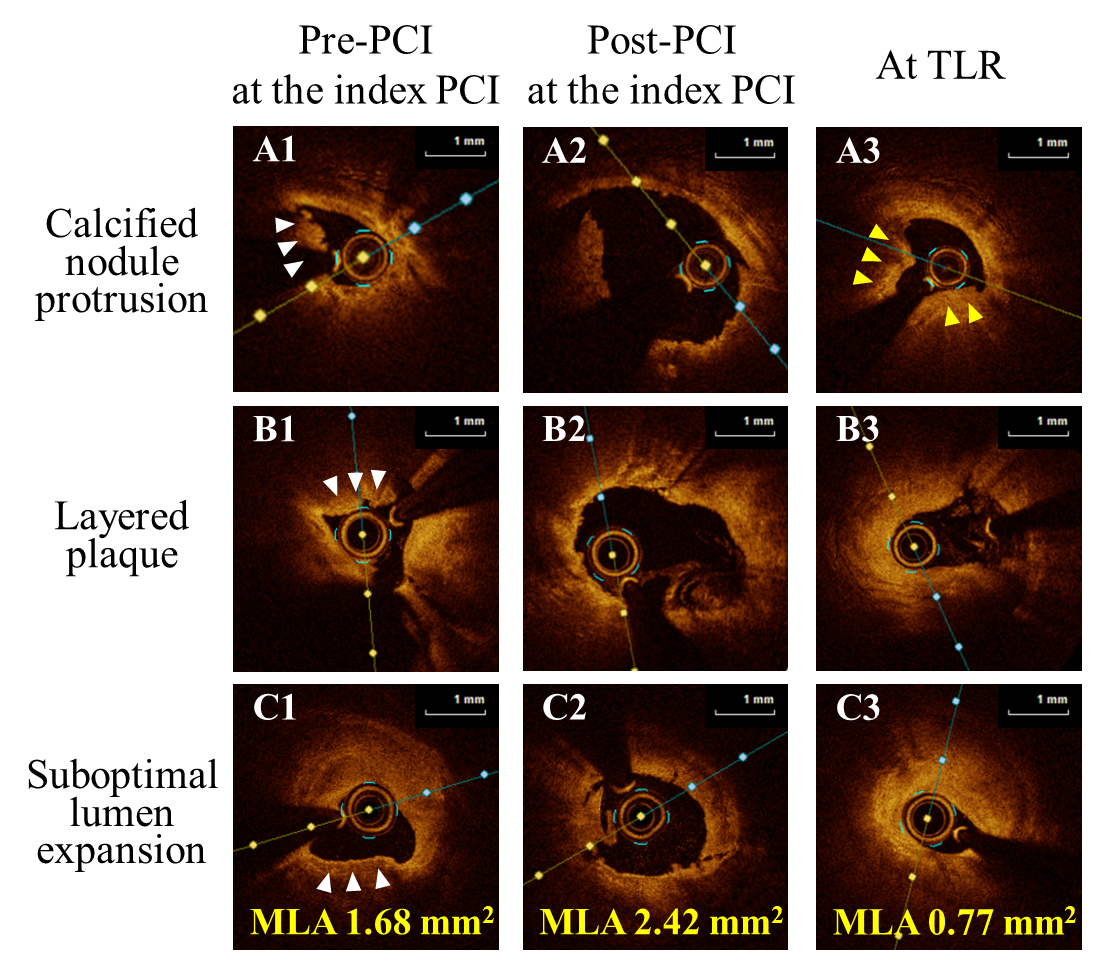

Supplement: Supplementary file 1 [file Image1.tif]
